# Optimized biomedical entity relation extraction method with data augmentation and classification using GPT-4 and Gemini

**DOI:** 10.1093/database/baae104

**Published:** 2024-10-09

**Authors:** Cong-Phuoc Phan, Ben Phan, Jung-Hsien Chiang

**Affiliations:** Department of Computer Science and Information Engineering, National Cheng Kung University, No. 1, University Road, Tainan City 701, Taiwan; Department of Computer Science and Information Engineering, National Cheng Kung University, No. 1, University Road, Tainan City 701, Taiwan; Department of Computer Science and Information Engineering, National Cheng Kung University, No. 1, University Road, Tainan City 701, Taiwan

## Abstract

Despite numerous research efforts by teams participating in the BioCreative VIII Track 01 employing various techniques to achieve the high accuracy of biomedical relation tasks, the overall performance in this area still has substantial room for improvement. Large language models bring a new opportunity to improve the performance of existing techniques in natural language processing tasks. This paper presents our improved method for relation extraction, which involves integrating two renowned large language models: Gemini and GPT-4. Our new approach utilizes GPT-4 to generate augmented data for training, followed by an ensemble learning technique to combine the outputs of diverse models to create a more precise prediction. We then employ a method using Gemini responses as input to fine-tune the BioNLP–PubMed–Bert classification model, which leads to improved performance as measured by precision, recall, and *F*1 scores on the same test dataset used in the challenge evaluation.

**Database URL**: https://biocreative.bioinformatics.udel.edu/tasks/biocreative-viii/track-1/

## Introduction

Biomedical named entity recognition (NER) and relation extraction (RE) play critical roles in drug discovery [1–3], clinical decision support⁠⁠ [4–6], and life science research by identifying and categorizing entities like genes, proteins, diseases, and drugs. These entities are key for information retrieval, literature curation, and knowledge extraction from vast unstructured biomedical data. To facilitate the research in these areas, the BioCreative VIII challenge [7]⁠⁠ calls for community effort to identify, categorize, and detect novel factors in relationships between biomedical entities in unstructured text. One of the challenge tracks, track 1: Biomedical Relation Extraction Dataset (BioRED), focuses primarily on NER and RE tasks. This track comprises two subtasks: Subtask 1 and Subtask 2. In Subtask 1, NER has already been performed, and participant teams are tasked with detecting the relationship between any pair of given entities, as well as the type of relation and novelty properties using the title and abstract text of PubMed articles. Subtask 2 is more challenging, as it requires teams to perform both NER and RE tasks using the only provided input of titles and abstracts from PubMed articles. Both NER and RE tasks present varying levels of difficulty, necessitating the use of distinct techniques and datasets.

**Figure 1.**
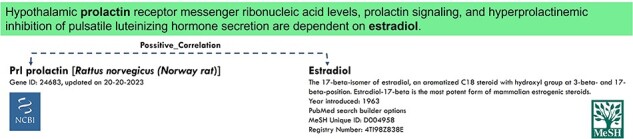
Example of NER and RE tasks with two entities annotated in different ontologies.

For NER tasks, there are different ontologies, such as “MeSH” [8]⁠⁠ for chemicals, “dbsnp” [9]⁠⁠ for variants, “NCBI Taxonomy” [10]*⁠⁠* for species, and “OMIM” [11]⁠ for diseases or genes, which are usually used to annotate entities with their entity identifiers. Figure 1 shows an example of NER annotation with two different ontologies. An entity identifier is a unique code or name for multiple entities sharing the same concept. For instance, both “Hepatocyte nuclear factor-6” and “HNF-6” are annotated with the entity identifier “3175” in NCBI Gene Ontology, and “End-stage renal disease” and “ESRD” share the entity identifier D007676 in “MeSH” [8]*⁠⁠*. There are several ways to categorize these entity identifiers, such as “PubTator” [12]⁠⁠ classifies them into “Gene,” “Chemical,” “Species,” “Diseases,” “Mutation,” and “CellLine,” while BioRED [13]⁠ modified the “Mutation to Variant.” There are other classes such as “Chromosome” and “Protein”. The NER task encounters three main challenges. First, entities are not always single words; they can be phrases containing special characters that intertwine with other punctuations in the sentence. Additionally, certain entities appear in abbreviated forms and may vary in wording or naming across different sections of the text. The NER system must identify these instances and treat them as representing the same concept. Second, an entity may belong to different ontologies depending on the context of the text being considered and thus have different entity identifiers. Therefore, the NER system needs to be able to recognize the appropriate ontologies before extracting the correct entity identifiers. Furthermore, NER tasks face multistage detection challenges, where entity identifier mapping is performed only after the entities have been detected and categorized into the appropriate ontology. The final challenge in the NER task is that there are numerous entity identifiers belonging to different ontologies, as mentioned earlier. Consequently, the NER cannot be developed using simple classification models alone.

Based on the NER results, the relations uncover connections through RE tasks, helping researchers understand complex biological and medical phenomena. These connections are categorized into types like “positive correlation,” “negative correlation,” “association,” “binding,” “drug interaction,” “cotreatment,” “comparison,” and “conversion,” as defined by BioRED data, based on the document’s context. Notably, an additional annotation factor known as “novelty” has gained attention and been introduced as a property of relations. It serves to indicate whether a relation is a well-established fact or a novel discovery, thus enhancing the utility of new knowledge extraction and mitigating redundancy in information. Identifying connections among entity identifiers often encounters numerous difficulties. When evaluating relationships, every conceivable pair of the *n* entity identifiers requires scrutiny, resulting in *n*(*n* − 1)/2 pairs to assess per document. Moreover, the precision of these procedures heavily relies on the accuracy of NER tasks and the contextual nuances within sentences housing these entities. Similar to NER tasks, the RE tasks may also encounter the problem of multistage prediction. In these tasks, the relation type detection tasks need to be done on top of relation detection, and the novelty prediction can be done after relation and relation types have been confirmed. Additionally, the small size of source training data and the unbalance in data quantity of various relation types cause critical challenges for text mining and natural language learning model construction.

In order to facilitate the aforementioned tasks and fulfill various NLP requirements in the field of biomedicine, numerous text corpora have been developed, accompanied by extensive research efforts [8, 14–17]⁠. Nevertheless, the majority of these methods focus on the relationship between two entities within a single sentence, even though numerous documents demonstrate that the actual relations can extend across multiple sentences. In addition, these research scopes focus only on specific domains, such as drug–drug interaction [14]⁠ and protein–protein interaction⁠, have not been evaluated on the context of other tasks. To the best of our knowledge, the most recent advancements in this field are BERT-GT [18] and BioREX [19] methods. BERT-GT operates by taking document abstracts with annotated entities and entity identifiers as input data, subsequently leveraging PubMedBERT for fine-tuning and predicting relations, relation types, and the novelty property. While BERT-GT [18] excels in the RE tasks, it does not encompass NER tasks. BioREX [19]⁠ offers enhanced RE performance, but it lacks NER task integration and does not include novelty prediction. On the NER task, AIONER [20]⁠ is a high-performing model for extracting biomedical entities, but it does not cover biomedical entity identifier identification (or entity normalization), a key element for RE tasks.

In an attempt to integrate the strengths of the mentioned methods and overcome their individual functional limitations—such as AIONER’s lack of entity identifier mapping, BERT-GT’s omission of NER tasks, and BioREX’s absence of novelty prediction—we introduce our multiple-model solution to comprehensively meet all the previously mentioned requirements. Additionally, we present a simple approach utilizing two well-known large language models (LLMs), Gemini and GPT-4, to demonstrate their effectiveness in biomedical text RE.

## Material and method

### Datasets and augmentation with GPT-4

For the development and evaluation of our system, we leverage data from the “BioCreative VIII” challenge. The Train dataset comprises 500 abstracts annotated with entities and relations for development, and the validation dataset has 1000 abstracts without annotation along with an additional 10 000 abstracts without annotation for the final evaluation phase. The data are in three formats: XML, JSON, and PubTator. Since the data used for training are much smaller than the data used for testing, we applied several steps by using GPT-4 as follows to augment datasets for further training.

At first, the original JSON training files were directly uploaded to GPT-4, ensuring precise interpretation and structural integrity of the data. However, our model necessitated input in the form of PubTator files, which is easier for implementation and validation. To address this, a subsequent dialog was initiated with GPT-4 to teach it the intricacies of converting data from the JSON format to the PubTator format. Due to capacity constraints of GPT-4, the datasets were divided into numerous small batches of 10 documents each to enhance processing efficiency and accuracy. Through iterative refinement, this process enabled GPT-4 to sharpen its understanding of the data fields in the provided dataset and comprehend the topological format of the input data.

The text generation is then powered by prompt engineering with GPT-4. Because GPT-4 is general LLM that can response to queries in many different domains such as politic, medical, math, and news, we add a step to instruct it focus on the scope of our training data (i.e. biomedical research papers). Typically, GPT-4 takes basic keywords like “English papers,” “Biomedical papers,” and “Abstract with title” and then produces foundational text as outcomes, which match to the training data characteristics.

Finally, the NER, RE, and novelty tasks have been done following the instruction in the guiding document (i.e. the “BioRED_Annotation_Guideline.pdf” file from challenge). Initially, this document was uploaded to GPT-4, followed by the rules of several prompts. These prompts were designed to gather explanations from GPT-4 regarding entity types, relation types, and the nuances of novelty. This step instructs the GPT-4 to understand the inner semantic structure of the JSON file and able to recognize the entity types, relation type, and the novelty property in the given text. Examples of such prompts include: “Please explain the definition of this type?” and “What kind of words will be used in the novelty?” Additionally, comparative inquiries were made, such as “What is the difference between this category and another category?” The responses generated by GPT-4 were then verified against actual information repeatedly. This iterative process aimed to enhance GPT-4’s understanding of the marking standards, ensuring that its annotations closely aligned with our intended objectives.

Besides the above-mentioned datasets and augmented data, we also used the other data from OMIM [11]⁠, MeSH [8], NCBI Gene [21], NCBI dbSNP [9]. These datasets include dictionaries of entity and entity identifier mapping text pairs, which are used for NER tasks in entity normalization. The Method description section will clarify this point in more detail.

### Method description

Our approach encompasses a comprehensive process, spanning from NER to RE and novelty prediction. This process can be divided into two key activities: NER and RE.

#### NER task

**Figure 2.**
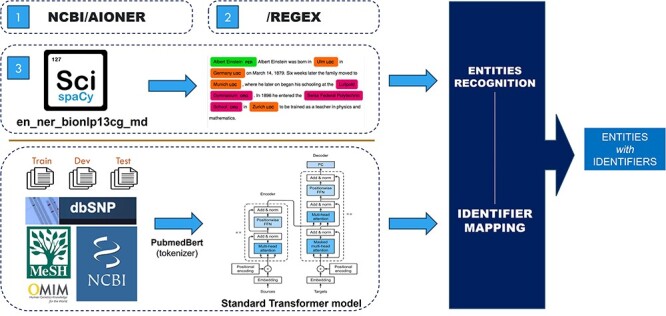
The overview of the NER structure.

The role of this task is to extract the biomedical words from a given text and then categorize these entities into entity types such as Diseases and Chemical. With the entity-type dataset, a standard transformer model is used to detect the entity identifier. Figure 2 shows the general view of our architecture of the three approaches we used.

##### Counting number of coexisting pairs by entity types


(1) $$P\left( {n,k} \right) = \frac{{{n_t}!}}{{\left( {{n_t} - k} \right)!}}$$

where *n_t_* is the number of entity type in our dataset. *k* is the number of entity types existing in the same document.

##### The probability score of each coexisting pair of entity types


(2) $$P = \left[ {p\left( {e{t_i},e{t_j}} \right)} \right] = \left[ {\left( {\frac{{\mathop \sum {e_i}}}{{{n_{ij}}}},\frac{{\mathop \sum {e_j}}}{{{n_{ij}}}}} \right)} \right],$$

where *et_i_* and *et_j_* are two entity types that coexist in the same document.

*e_i_* and *e_j_* are the corresponding number of entities in *et_i_* and *et_j_*. *p*(*et_i_, et_j_*) is the probability of coexistence pair between *et_i_* and *et_j_*. *n_ij_* is the number of documents that contain both *et_i_* and *et_j_*.

##### Fulfillment score calculation


(3) $$ fullfillmentScore(a,\ docA) = \frac{{{{\mathop \sum }_{e{t_i} \in docA}}{\ }\frac{{\left| {e{t_i}} \right|*P\left( {e{t_i},et\left( a \right)} \right)\left[ 1 \right]}}{{P\left( {e{t_i},et\left( a \right)} \right)\left[ 0 \right]}}}}{{\left| {docA\left( {e{t_i}} \right)} \right|}}$$

where *a* and *et*(*a*) are the entity and its type considered to add into the entity list of doc*A*. doc*A* is the specific document that needs to add entity *a*. *et_i_* is the entity type that exists in doc*A*. *P*(*et_i_*, *et*(*a*)) [0] and *P*(*et_i_, et*(*a*)) [1] are the left and the right values of the table *P* at the pair (*et_i_, et*(*a*)). |doc*A*(*et_i_*)| is the number of entity type in doc*A*.

##### Approach 1

This approach uses “SciSpacy” [22] with the “en_ner_bionlp13cg_md” model for entity extraction, followed by a classifying model to match the entity types and identifiers using training data and data from “MeSH” [8]⁠⁠, “OMIM” [11]*⁠*, “Mutation2PubTator” (from “PubTator FTP” [23]), “Cellosaurus” [24], and “Species2PubTator” (from “PubTator FTP” [22]). These data are presented as sequences of entity texts matched to corresponding sequences of identifiers. Because of the large number of identifiers in each entity type, the standard transformer is used instead of normal classification methods for mapping the entity to its identifier. Specifically, the default transformer is trained with entities as input and identifier as output. With this approach, we accept the lack of input context and solve the issue by a probability score table, which will be presented in the next session.

##### Approach 2

This approach utilizes “AIONER” [20]*⁠* with fine-tuning to extract entities from input text then applies the Approach 1 model for identifier information extraction.

##### Approach 3

This approach uses regular expressions to identify existing entities in the training data, eliminates duplicates, and stores the results as a dictionary file for filtering incoming text. Specifically, RegEx is used to extract nouns and noun phrases based on their parts of speech and keep them into a dictionary data structure file. For example, a simple regression expression of {< JJ.*|JJ*>*< NN.*>} can extract the entity “seasonal affective disorder” (document “BC8_BioRED_Task1_Doc8” in training data file).

Combining any of the two options can lead to many duplicated entities with distinct types. For instance, the entity “ArsB” can occur as both a “Chemical (C581941)” entity and a “Gene (OMIM: 611542)” entity. To solve this problem, we created a probability model based on entity-type co-occurrence within documents. This model includes two main components: a “probability score table” and a “fulfillment method”:

Probability score table: This set represents the probability scores of co-occurrences between entity types in pairs, calculated by the average number of entities of each type found in the same document. Generally, the number of pairs in Table *P* is calculated by the number of permutations of the number of entity types, which is depicted in Equation (1). In this paper, we set *k* = 2, and is open for further improvement in the future with more ablation studies, meaning only the co-occurrence of two entity types are concerned in our system. The score of each probability in *P* is computed using Equation (2).

Fulfillment method: Equation (3) is used to calculate the fulfillment score based on the probability table. Each document has a finite set of the entity list from merging between three NER approaches defined above. An entity would be added to the entity list of a specific document that has a greater fulfillment score.

**Figure 3.**
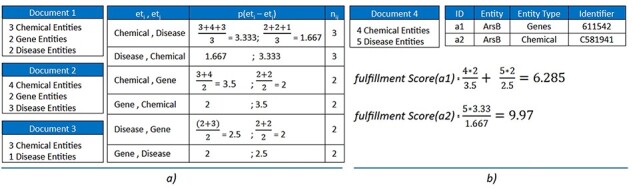
(a) Construct the probability table for three sample documents with *k* = 2. (b) Calculate the fulfillment scores for two sample entities: *a*1 and *a*2. The results show that *a*2 is preferred over *a*1.

Figure 3 shows an example of three sample documents with three entity types, six possible pairs of entity types. Three documents contain Chemical–Disease coexistence, while two documents contain only Chemical–Gene or Gene–Disease. Consider a new document (“Document 4”) that has two identical entities “ArsB” marked as *a*_1_ and *a*_2_ with different entity types of Genes and Chemical (and different identifiers 611542 for Genes and C581941 for Chemical). The “fulfillment score” is calculated for both cases, and the results show that the score returned for *a*_2_ is higher than *a*_1_. The *a*_2_ would be added to the entity list of “Document 4,” where “ArsB” is annotated with identifier C581941 for “Chemical.”

#### Relation extraction

**Figure 4.**
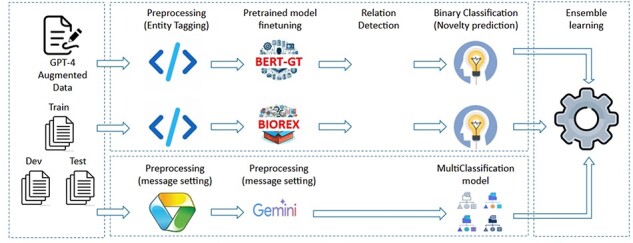
The flowchart of RE and novelty prediction method structure.

The purpose of the RE task is to ascertain potential connections between pairs of identifiers identified by NER tasks. Our methodologies involve extracting all potential identifier pairs from NER results and assessing them for relationships through three approaches: utilizing Gemini prompt with a classification model, fine-tuning pretrained models, and employing ensemble learning with existing models. The overview of the system is depicted in Fig. 4

##### Gemini prompt with classification model

Gemini is a free pretrained LLM available in both web and API versions, and it can be fine-tuned for specific tasks. However, directly fine-tuning the Gemini model with our custom data for classification tasks is resource-intensive and less flexible, especially given our limited training data. Instead, we use the text generated by Gemini as input for our custom classification model. In this approach, we engage the Gemini API (“version gemini-1.0-pro”) by submitting requests that contain both the title and abstract as context, along with a query question, to determine the existence of any relationship between each pair of entities. The responses obtained from Gemini are free-form text corresponding to all pairs within each document. To do this, we implement two message setting steps. In the first message setting step, create prompt messages based on the content of document (i.e. title and abstract). The structure of prompt messages is constructed as the following structure:

Given {title}. {abstract}. Is there any relation between {entity1 text} ({entity1 type}) and {entity2 text} ({entity type}).

Even though the queries are closed questions, Gemini responses are free text answers that contain rich information for explanation. As the results, 75 339 response text messages are collected by using 500 documents in the training dataset for this step. The second message setting step is to combine the response text from Gemini with the ground truth of relations within these documents to create a supervised dataset for a classification model. This dataset consists of 15 labeled classes, representing combined information on relation type and novelty criteria with one added class “Not found” to represent no relation, as detailed in Table 1. Utilizing this dataset, we construct the classification model with two linear layers and a softmax output, intending to estimate the probability of the input text belonging to a specific class. The tokenizer is loaded from “Microsoft/BiomedNLP-BiomedBERT-large-uncased-abstract” [25] via the “HuggingFace” collaboration platform.

**Table 1.** Gemini-responded data count by relation types

Label class	No. of item
Not found	61 691
Association, novel	5050
Association, no	2129
Positive_Correlation, novel	2823
Positive_Correlation, no	3764
Negative_Correlation, no	2300
Negative_Correlation, novel	1655
Cotreatment, novel	72
Bind, no	114
Cotreatment, no	26
Comparison, novel	88
Bind, novel	45
Conversion, no	3
Comparison, no	11
Drug_Interaction, novel	37
Drug_Interaction, no	8

##### Fine-tune pretrained models

These models leverages the techniques from BERT-GT [18] and BioREX [19] papers, in which the Microsoft/BiomedNLP-PubMedBERT-base-uncased-abstract is exploited for the tokenizer and prediction, then trained on biomedical relation training data in different ways. Since these methods have trained on large PubMed abstracts, reusing these methods helps to save much training effort. In the fine-tuning phases of these models, input data are preprocessed using tagging, wherein open and close tags are applied to each entity within the document. The tagging procedure enriches the information for both entity recognition and relationship identification. Additionally, this tagging approach facilitates the definition of relationships by incorporating both the source and target information within the tag names. For instance, consider “Example 1” below, where “maleate” identified as a Chemical entity and “claudin-2” as a Gene entity are processed through tagging:

Tubular proteinuria and oxidative stress were induced by a single injection of *@ChemicalEntitySrc$* maleate *@/ChemicalEntitySrc$* (400 mg/kg) in rats. *@ChemicalEntitySrc$* Maleate-induced *@/ChemicalEntitySrc$* renal injury included increase in renal vascular resistance and in the urinary excretion of total protein, glucose, sodium, neutrophil gelatinase-associated lipocalin (NGAL) and N-acetyl b-D-glucosaminidase (NAG), upregulation of kidney injury molecule (KIM)-1, decrease in renal blood flow and *@GeneOrGeneProductTgt$* claudin-2 *@/GeneOrGeneProductTgt$* expression besides of necrosis and apoptosis of tubular cells on 24 h (Example 1)

The models trained using these methods undergo fine-tuning with augmented data from GPT-4, followed by utilization in a classification prediction model. Our classification model employs nine labels, corresponding to eight relation types, with an additional label for “No Relation” indicating cases where no relation exists between the considered entity pairs. Furthermore, each model incorporates a filter set at a probability threshold of .7 for relation selection output to enhance relation accuracy. However, neither of these approaches include novelty prediction. To address this aspect, an additional classification step is introduced. Specifically, the chosen relations from each model are then fed into a novelty prediction process. This process includes a binary classification model utilizing the pretrained “alvaroalon2/biobert_diseases_ner” for both tokenization and classification, which aimed at determining the novelty of each relation.

##### Ensemble learning

After testing the described models and evaluating the process separately, we observed that each model performed well on different subsets of the dataset. To address this, we used an ensemble learning technique to combine the suggestions from all models, resulting in improved overall performance. As described in Fig. 4, our final approach merges all models mentioned earlier to achieve the final prediction step (detected relations with predicted novelty properties). Initially, the *F*1 scores from the challenge results are used as the weights for different systems in majority voting. Each member model output is submitted separately to Codalab Leader Board to get the evaluation results. At the entity pair types (e.g. “Gene–Gene” and “Chemical–Disease”), the highest performance score is selected as the weight for the model for that pair type. As the result, the highest *F*1 for each entity pair type among models is voted to return as the final output. These steps of submitting and selecting were repeated several times to improve performance of all entity pairs, and so improve the performance of stages.

## Results and discussion

The BioCreative VIII participant teams were allowed to submit five runs for each task, with each run containing the predicted results from a different method. Because the final results have several metrics and evaluated through many stages, there was no single best run for each team across all metrics. Instead, the highest score among submitted runs on each stage was reported along with the submitted run number [7].

The evaluation of both NER and RE tasks relies on three standard metrics: precision, recall, and *F*1 scores. Given the distinct stages within each task, performance assessment was conducted cumulatively across these stages. Specifically, the NER task has two evaluation levels: entity recognition level and identifier mapping level, while the RE task has four evaluation levels: entity pair levels, entity pairs with relation type level, entity pairs with novelty level, and entity pairs with both relation type and novelty property prediction levels. There are two benchmark datasets used in our evaluation: validation dataset and test dataset, which are available in the BioRED evaluation leader board at “Codalab” (https://codalab.lisn.upsaclay.fr/competitions/16381#results). Evaluations on these datasets are blind tests (the grown truth data and the evaluation source code are controlled by organizers). In this leader board, our performance was shown under account “peterphancong.”

With the validation dataset, our method achieves the best performance in comparison with other teams across all metrics and evaluation levels. Specifically, Table 2 shows that our performance is at the top performance compared to the other teams. Moreover, when compared to the next highest team, our results show a relative improvement of 23.5% in the last evaluation stage, 12.9% in the entity pair with novelty stage, 9% in the entity pair relation stage, and 3.8% in the entity pair stage.

**Table 2.** *F*-Score evaluation on the validation dataset shows that our method results in bold is outperformed the other existing methods

Method	Entity pair (%)	Entity pair|relation type (%)	Entity pair|novelty (%)	Entity pair|relation type|novelty (%)
Our method	**81.93**	**70.55**	**76.79**	**68.25**
richardjonker	78.93	64.18	67.99	55.27
mjsarol	78.20	65.30	66.49	55.22
tiagoalmeida	77.76	64.10	67.19	55.37

For NER task evaluation executed on the test dataset, we prepared three studies to analyze the results as follows:

- Run 1: use our approach to extract entities and transformer to match identifiers with enhancement provided by our probability model.
- Run 2: use PubTator to extract entities and transformer to match identifiers.
- Run 3: use AIONER [20] and transformer to extract entities and match identifiers.

Table 3 shows the result of this ablation study results, and Table 4 shows the results of other teams in the BioCreative VIII challenge. These results can be found in the report from organizers [7], where our team is numbered as 118. Specifically, using PubTator [12] is better than AIONER [20]⁠, and it is even improved with applying our probability model.

**Table 3.** The ablation study showing the effectiveness of using the probability model on the test dataset on Recall and F1 scores. The highest values among the runs are indicated in bold

	Entities recognition			Identifier mapping		
Run	*P* (%)	*R* (%)	*F* (%)	*P* (%)	*R* (%)	*F* (%)
Run 1	**83.34**	73.86	**78.31**	**77.76**	73.56	**75.61**
Run 2	60.02	79.98	68.58	52.68	**78.44**	63.03
Run 3	61.41	**89.9**	72.97	49.25	70.21	57.89

**Table 4.** The comparison using F (%) between our method and the other teams during challenge. The highest values are indicated in bold

Team	Entities recognition (*F*, %)	Identifier mapping (*F*, %)
156	**89.26**	**84.07**
129	78.58	76.35
127	78.30	75.98
Our results (“Team 118 in challenge”)	78.31	75.61
157	79.39	75.35
138	65.12	52.48
111	72.90	65.75
148	87.28	42.67
154	69.98	29.58

In our RE task evaluation executed on the test dataset, we conduct ablation studies to assess the performance of each individual method compared to ensemble learning models and our previous best runs from the challenge. Detailed results are presented in Table 5, while Table 6 provides a comparative analysis between our team’s efforts and those of other participants in the challenge.

**Table 5.** The ablation study results between individual methods and ensemble learning on the test dataset. The values in bold indicate the highest values among methods

	Entity pair			Entity pair|relation type			Entity pair|novelty			Entity pair|relation type|novelty		
Method	P	R	F	P	R	F	P	R	F	P	R	F
Median score of our previous submissions (November 2023)	***77.93***	69.65	73.56	51.64	54.79	53.17	52.97	60.42	56.45	**41.61**	39.88	40.73
BioREX	70.90	60.60	65.30	45.80	39.02	42.20	44.4	38.00	41.00	27.30	23.30	25.10
BERT-GT + GPT-4 data	72.00	77.70	74.20	50.30	53.60	51.90	54.4	58.00	56.20	37.70	40.10	38.90
Gemini	68.94	58.96	63.56	45.09	38.67	41.63	43.22	37.31	40.05	26.99	23.33	25.03
Ensemble learning (new)	69.97	**81.64**	**75.36**	**51.70**	**60.52**	**55.76**	**53.66**	**62.89**	**57.91**	40.17	**47.20**	**43.41**

**Table 6.** Performance comparison using *F* (%) for the best run of all participant teams during challenge. The values in bold indicate the highest values among methods

Team	Entity pair (*F*, %)	Relation type (*F*, %)	Entity + Novelty (*F*, %)	All
129	75.59	56.67	**59.22**	44.41
114	74.27	55.05	58.54	43.5
156	**77.17**	**58.95**	58.27	**44.55**
Our upgraded results	75.36	55.76	57.91	43.41
127	75.38	55.93	57.69	43.04
Our results	73.46	55.31	57.32	43.49
138	74.08	53.31	56.85	40.73
142	74.28	52.58	56.26	38.91
157	73.56	52.76	56.05	39.71
116	56.41	26.54	40.94	18.26
148	52.67	38.02	39.46	28.35
155	48.96	28.78	30.37	17.61
111	24.2	8.66	15.48	5.33
154	32.48	7.27	–	2.96

## Conclusion

In the BioCreative VIII challenge, organizers played a key role in defining and consolidating entity and relation types, as well as introducing the novelty property, which is crucial for estimating the value of detected relations. The challenge also provided rich NER and RE datasets for system development and evaluation. This paper describes our team’s method with an innovative approach for this challenge. Compared to previous submission in the BioCreative VIII challenge, our new method demonstrates the effective use of LLMs in two areas of data augmentation and model enhancing, which contribute to the final performance in the combination with the other member models using ensemble learning. Despite our progress, performance levels are not yet considered trustworthy for practical applications, and there are numerous other potential LLMs in the biomedical field, indicating ample room for improvement. In addition, prompt engineering does not apply adequately to enrich the output and enhance accuracy. These issues will be investigated and continued to upgrade in the future.

## Data Availability

It is available on the BioCreative VIII website. After the challenge has finished, the organizers put the data to evaluate in CodaLab. For details, please visit the following link: https://biocreative.bioinformatics.udel.edu/tasks/biocreative-viii/track-1/.
